# Contribution of cytokeratin 19-expressing cells towards islet regeneration induced by multipotent stromal cell secreted proteins

**DOI:** 10.1093/stmcls/sxaf036

**Published:** 2025-06-10

**Authors:** Nazihah Rasiwala, Gillian I Bell, Anargyros Xenocostas, David A Hess

**Affiliations:** Department of Physiology and Pharmacology, Western University, London, ON N6A 3K7, Canada; Molecular Medicine Research Laboratories, Robarts Research Institute, London, ON N6A 5B7, Canada; Department of Hematology, London Health Sciences Center, London, ON N6A 5W9, Canada; Department of Physiology and Pharmacology, Western University, London, ON N6A 3K7, Canada; Molecular Medicine Research Laboratories, Robarts Research Institute, London, ON N6A 5B7, Canada

**Keywords:** multipotent stromal cells, mesenchymal stem cells, islet regeneration, diabetes, lineage tracing, ductal cell endocrine progenitors

## Abstract

Residual beta cell function has been documented in “medalist” patients who have lived with Type 1 diabetes (T1D) for >50 years. In addition, endocrine cell neogenesis first occurs in the developing human embryo from progenitor cells derived from pancreatic ductal epithelial structure. Thus, beta cell conversion from a dormant epithelial precursor remains a promising approach to regenerate islets during T1D. We have previously shown that intra-pancreatic (iPan) injection of Wnt pathway-stimulated conditioned media (Wnt+ CdM) generated from human bone marrow-derived multipotent stromal cells (MSC) contained islet regenerative factors that reduced hyperglycemia and recovered beta cell mass in streptozotocin-treated mice. However, the endogenous source of regenerated beta cells remains unknown. Herein, we employed cytokeratin 19 (CK19)-CreERT Rosa26-mTomato lineage-tracing mice to assess the endocrine conversion of CK19+ cells during MSC CdM-induced islet regeneration. Mice iPan-injected with Wnt+ CdM demonstrated reduced blood glucose levels and improved glucose tolerance compared to mice injected with unconditioned basal media. CdM-injected mice also showed increased islet number and beta cell mass, as well as CK19+ cells within regenerating islets. The frequency of insulin + cells that co-expressed tdTomato within dissociated pancreas samples observed via flow cytometry was 5-fold higher in Wnt+ CdM-injected mice (~5%) compared to basal media-injected controls (~1%). Collectively, in vivo lineage tracing revealed conversion of CK19+ cells to functional beta cells partially contributed to islet regeneration induced by Wnt-activated MSC CdM. Future studies are required to delineate alternate cell types and mechanisms participating in islet regeneration induced by direct delivery of MSC-CdM.

## Graphical Abstract

**Graphical Abstract:**
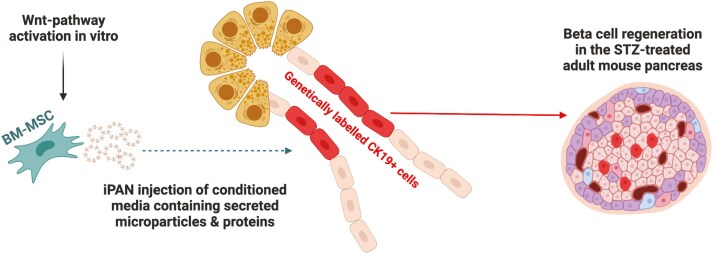

**Significance statement**

We employed cytokeratin 19-CreERT Rosa26-mTomato lineage-tracing mice to assess endocrine conversion of CK19+ cells during islet regeneration induced by mesenchymal stem cell conditioned media (MSC CdM). Mice injected with MSC CdM demonstrated reduced blood glucose, improved glucose tolerance, and increased beta cell mass compared to mice injected with unconditioned basal media. The frequency of insulin-expressing cells that co-expressed tdTomato within dissociated pancreas samples observed via flow cytometry was 5-fold higher in MSC CdM-injected mice compared to basal media-injected controls. Conversion of CK19+ cells to functional beta cells contributed to islet regeneration induced by MSC CdM.

## Introduction

The origins of newly formed pancreatic beta cells in the adult endocrine pancreas remain elusive. Although induction of beta cell regeneration combined with protection from subsequent autoimmune destruction has been proposed as a curative therapy for type 1 diabetes (T1D), a better understanding of the mechanisms underlying islet regeneration is required in the quest to develop an effective regenerative therapy for diabetes. T1D is a chronic autoimmune disorder characterized by T-lymphocyte-mediated destruction of insulin-secreting pancreatic beta cells and results in the inability to regulate blood glucose levels.^1^ Over the past two decades, epidemiological studies conducted in the United States,^2^ Canada,^3^ China,^4^ the United Kingdom, and other European countries,^5^ have reported a steady increase in the prevalence of T1D. This alarming trend highlights a pressing need for innovative therapeutic approaches to better manage and treat T1D. Individuals living with T1D also face challenges such as extreme glucose variation, lower health-related quality of life, and premature development of severe cardiovascular and renal comorbidities,^6-8^ underscoring the urgency for the development of curative interventions. Consequently, efforts dedicated to uncovering the mysteries of beta cell regeneration may pave the way towards transformative regenerative therapies that alleviate the burden of T1D and improve the lives of millions affected by this debilitating condition.

Beta cells lost due to T1D can be regenerated in situ. In rodent models of pancreatic damage using beta cell ablation via streptozotocin (STZ) or alloxan treatment, beta cell regeneration was evident as early as 10 days post-injury.^9-12^ In humans, evidence of beta cell regeneration has been limited to studies analyzing post-mortem pancreas samples from patients with T1D. In the “medalist” study, histological analyses on pancreas tissue from T1D patients that were insulin-dependent for >50 years duration consistently demonstrated residual clusters of insulin-positive cells in pancreas sections and c-peptide detection in serum, indicating beta cell regeneration by endogenous mechanisms was concealed by continual and unrelenting autoimmunity.^13,14^ Analyses of pancreata from children with T1D uncovered higher rates of beta cell proliferation compared to age-matched controls,^14^ and recent-onset individuals with T1D showed increased alpha and beta cell proliferation in islets with ongoing insulitis.^15^ However, in the pancreas of adults with established T1D, rates of beta cell proliferation were lower than non-T1D controls, suggesting that early diagnoses combined with beta cell regeneration strategies may provide a unique window to slow or stop T1D progression.

Multipotent stromal cells (MSC), also referred to as mesenchymal stem cells, represent a versatile population of progenitors from the mesodermal lineage distributed throughout various tissues in association with blood vessels. Human bone marrow (BM)-derived MSC has garnered significant attention for regenerative medicine application to mediate tissue repair and address autoimmunity due to the unique capacity to dampen immune responses and induce immune tolerance.^16^ MSC possess the ability to home to sites of tissue damage and orchestrate endogenous repair processes through the release of regenerative proteins and other bioactive molecules packaged within extracellular vesicles,^17,18^ thereby promoting regeneration and functional recovery. Therefore, MSC-derived secreted factors offer promise in the development of “cell-free” regenerative strategies for autoimmune disorders that mediate tissue damage or dysfunction. By exploiting the MSC-secretory capacity of regenerative proteins and immunomodulatory effectors, we aim to develop innovative therapeutic strategies that circumvent challenges associated with cell transfer, allowing for more accessible and effective interventions in regenerative medicine for T1D.

Our group and others have demonstrated the therapeutic potential of transplanted BM-MSC to stimulate islet regeneration.^19-22^ In immunodeficient mice rendered hyperglycemic via STZ treatment, improvements in glycemic control, glucose tolerance, and insulin secretion, alongside increased beta cell mass were observed after transplantation with BM-MSC.^19-22^ A notable caveat was that BM-MSC demonstrated donor-dependent variability in the ability to reduce glucose levels.^19,23,24^ We used mass-spectrometry-based proteomics to identify that multiple differentially expressed regenerative proteins upregulated in regenerative versus nonregenerative MSC.^24^ Subsequently, we used quantitative, label-free proteomics and machine learning to prospectively screen for beta cell regenerative potency in 30 donor-derived BM-MSC lines. We identified 16 proteins within human MSC-generated conditioned media (CdM) that effectively predicted islet regenerative capacity.^25^ BM-MSC lines with a heightened capacity to lower hyperglycemia generated a secretome that showed an increased abundance of cytokines upregulated during active Wnt pathway signaling.^25,26^ Exogenous stimulation of canonical Wnt/beta-catenin signaling using the glycogen synthase kinase-3 inhibitor CHIR99021 exposure in culture, not only helped mitigate observed donor-specific variability but also restored regenerative capacity in BM-MSC that did not previously promote islet regeneration.^19,25,26^ Ultimately, intrapancreatic (iPan)-delivery of concentrated Wnt pathway-stimulated MSC conditioned media (Wnt+ CdM) into STZ-treated hyperglycemic mice improved glucose regulation and increased beta cell mass without the requirement for MSC transplantation.^27^ Indeed, Wnt+ CdM-injection set in motion a cascade of events consistent with the emergence of small functional islets associated with ductal structures. However, we have yet to identify the cellular mechanisms driving the increase in beta cell mass and, to date, no studies have traced the origin of new beta cells formed during Wnt+ CdM-stimulated beta cell regeneration.

Sources of newly formed beta cells in the adult pancreas have been extensively debated. Conflicting results generated using different forms of beta cell ablation in lineage-tracing mice underscores the complex regulation of islet regeneration via multiple mechanisms. Several distinct in vivo linage tracing models have identified alpha,^28-33^ acinar,^34,35^ ductal epithelial,^36-41^ and pre-existing beta cell division,^42,43^ as potential precursors that participate in islet regeneration. In Kuljanin et al,^27^ pancreas sections from mice injected with Wnt+ CdM uniquely contained cytokeratin 19 (CK19) + cells, a ductal epithelial cell marker, within regenerating islets, and an increased percentage of regenerating islets were in direct contact with hyperplastic CK19+ ducts.^27^ Therefore, we hypothesized that islet regeneration induced by Wnt+ CdM-injection followed a mechanism analogous to neoislet formation in the developing mammalian pancreas, where islets sprout from the pre-established ductal network. This study utilized CK19-CreERT Rosa26-mTomato mice with STZ-induced beta cell ablation to trace the contribution of CK19+ cells towards a neogenic beta cell phenotype induced by iPan-injection of Wnt+ CdM.

## Materials and methods

### MSC culture and condition media generation

Primary human BM-MSC (*N* = 8) were cultured until passage 4 in AmnioMax complete media + proprietary supplement containing 15% FCS (Thermo Fisher Scientific, Waltham, MA). When MSC reached 80% confluence, MSC was washed twice with PBS to remove residual growth factors and serum and treated with either 10 μM CHIR99021 (AbMole Biosciences) to activate Wnt-signaling, or with DMSO as vehicle control (Untreated CdM) in basal AmnioMax media without supplement. After 24 hours incubation, CdM was collected and concentrated (~20-fold) in 3 kDa filter spin columns centrifuged at 4100*g* for 90 minutes at 4°C. Concentrated CdM was normalized to 0.1-0.25 ug total protein per uL used within 24 hours or cryopreserved at −80°C for later use.

After CdM collection BM-MSC were confirmed as >90% viable, >95% expressed CD73+ (344008, BioLegend), CD90+ (328108, Biolegend), and CD105+ (323224, BioLegend) and <1% expressed CD34 (562577, BD Biosciences) or CD45 (304016, BioLegend). A subset of BM-MSC from each line was permeabilized to detect intracellular beta-catenin levels (53-2567-42, Invitrogen) analyzed on an LSRII flow cytometer (BD Biosciences). To confirm nuclear beta-catenin upregulation within MSC, beta-catenin protein was quantified after cytoplasmic versus nuclear protein fractionation (Boster Bio) using the human beta-catenin SimpleStep ELISA kit (Abcam).

### Beta cell ablation, iPan injection, and glucose monitoring

All animal experiments followed guidelines published by the Canadian Council for Animal Care, and the animal protocol (AUP 2018-184) was approved by the Animal Care Committee at Western University, Canada. CK19-CreERT Rosa26-mTomato mice were generated by crossing homozygous CK19-CreERT mice (Jackson Labs, Bar Harbor, ME #026925)^44^ with homozygous Rosa26-mTomato reporter mice on a C57BL/6J;129S6 mixed background (Jackson Labs #007905).^45^ CK19-CreERT Rosa26-mTomato mice (8-10 weeks old, female and male) were treated with 6 mg of tamoxifen by oral gavage on 2 consecutive days to induce permanent tdTomato expression. Five days after tamoxifen treatment, mice were given 50 mg/kg STZ i.*P*. for 5 consecutive days (days 0-4). Hyperglycemic mice with non-fasted blood glucose of 12-25 mmol/L, were treated either on day 10 (short-term monitoring) or day 14 (long-term monitoring) using a single iPan-injection as previously described.^23,27^ Briefly, anesthetized mice were shaved and the pancreas was exposed through a 1 cm incision. A total volume of 20 μL of Wnt + CdM or Untreated CdM containing a total of 2-5 ug secreted protein or basal media was injected directly into the pancreas and the incision was sutured. Mice were weighed and non-fasted blood glucose was measured twice weekly between 8:00 and 10:00 a.m. using a FreeStyle Lite glucometer (Abbott Diabetes Care). For glucose tolerance tests, mice were fasted for 4 hours, i.p.-injected with sterile glucose bolus (2.0 g/kg) and blood glucose was measured by tail vein puncture at 0, 5, 10, 15, 30, 60, 90, and 120 minutes post-injection.

### Immunohistochemical and immunofluorescent analyses

Pancreas tissue from each mouse was excised, weighed, snap-frozen in optimal cutting temperature media (Tissue Tek; Sakura-Finetek) at -30°C, and cut into 12 µm sections. For insulin+ islet number and beta cell mass analyses, sections were stained using rabbit anti-insulin (ab181547, Abcam) and 3,3′-diaminobenzidine as previously described^19^ and imaged using brightfield microscopy. Beta cell mass was calculated by: insulin+ cell area / total area × pancreas weight in 3 sections per mouse > 150 µm apart. Immunofluorescent analyses were performed using rat anti-CK19 (TROMA-III, DSHB, Iowa City, IA), rat anti-acinar cell (target referred to as Mpx1 epitope^46^) (MABS2144, Sigma, Burlington, MA), rabbit anti-insulin (ab181547, Abcam), and/or mouse anti-glucagon (ab10988, Abcam) antibodies followed by DAPI nuclear co-staining, to quantify beta/alpha cell ratio, islet-duct association, and frequency of traced tdTomato+ insulin+ cells. Beta/alpha cell ratio was calculated by: insulin+ islet area/glucagon + islet area and was averaged for each islet observed in 3 sections per mouse > 150 µm apart. To determine islet-duct association, each islet in 3 sections per mouse was designated as either in direct contact with or not in direct contact with CK19 + ductal structures. To quantify the frequency of tdTomato + beta cells, total number of insulin+ tdTomato+/total insulin+ cells were counted in 3 sections per mouse. All quantification was conducted in a blinded fashion using QuPath software.^47^

### EdU injection and quantification of islet cell proliferation

Immediately following iPan-injection, separate cohorts of mice were i.p.-injected with 0.25 mg 5-ethynyl-2′-deoxyuridine (EdU) for 3 consecutive days. Pancreata were collected 24 hours following the final EdU-injection. Pancreas cryosections were stained for insulin, incubated with the EdU detection cocktail as per manufacturer’s instructions (Invitrogen), followed by DAPI costaining. To quantify the frequency of proliferating beta cells for each mouse, the total number of EdU+/insulin + co-positive cells and the total number of insulin + cells were counted for each islet identified in 3 sections per mouse.

### Flow cytometry on pancreas tissue

All incubations were conducted at room temperature unless otherwise stated. Pancreas samples were dissected laterally to collect both head and tail components. Each section was manually cut into <1 mm pieces prior to digestion using collagenase V (Sigma) for 20 minutes at 37°C followed by TrypLE (ThermoFisher Scientific) for 5 minutes at 37°C. Digested pancreas tissue was passed through a 40 µm cell strainer to establish a single cell suspension. Cells were incubated with Zombie Yellow viability dye (BioLegend) for 15 minutes prior to fixation with 10% formalin for 5 minutes. and permeabilization with 1% Triton X-100 for 15 minutes. Cells were incubated with the following antibodies for 1 hour: CK19 (AF488, ab192643, Abcam), insulin (AF647, 565689, BD Biosciences), and rat anti-acinar cells (MABS2144, Sigma, secondary antibody conjugated to FITC). Samples were analyzed on an LSRII flow cytometer (BD Biosciences) and all flow cytometry data was analyzed using FlowJo software.

### Statistics

All data was expressed as mean ± standard deviation (SD). Non-fasted blood glucose levels and glucose tolerance test curves were analyzed using a repeated measure two-way analysis of variance (ANOVA). All other parameters were analyzed by one-way ANOVA followed by Tukey’s multiple comparison test. Significance between groups was determined as *P < 0.05, **P < 0.01, or ***P < 0.001. Data was analyzed and graphed using GraphPad Prism version 9.

## Results

### CK19+ cell tracking in CK19-CreERT Rosa26-mTomato mice

**Figure 1.**
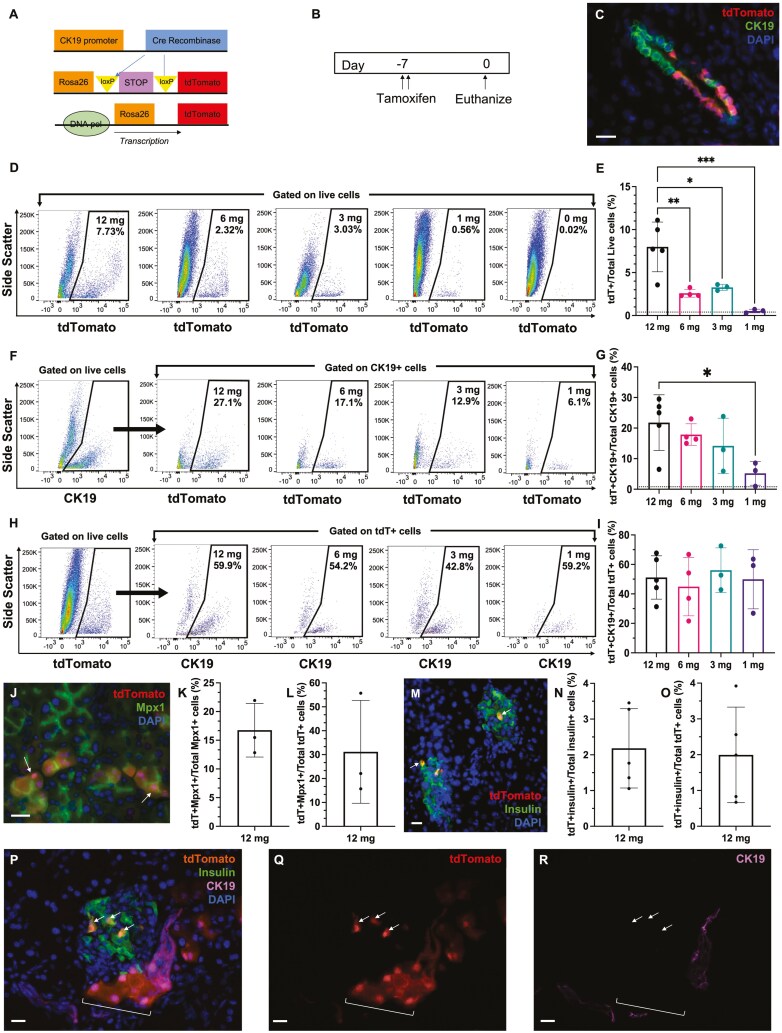
Characterization of CK19+ cell tracing in CK19-CreERT Rosa26-mTomato mice. (A) The CK19-CreERT mouse was crossed with Rosa26-mTomato reporter mice to trace CK19+ cells during islet regeneration induced by MSC CdM. (B) Mice were treated with 12 mg (6 mg per day for 2 days; *n* = 5), 6 mg (*n* = 4), 3 mg (*n* = 3), or 1 mg (*n* = 3) tamoxifen or corn oil vehicle (*n* = 3) and analyzed for tdTomato-labeling 1 week later. (C) Representative image of mosaic tdTomato-labeling in a CK19+ duct at 7 days post-tamoxifen injection. (D) Representative flow cytometry plots of viable tdTomato+ cells from the pancreas of mice 7 days after injection with tamoxifen or corn oil vehicle. (E) Injection of 12 mg tamoxifen resulted in the highest frequency of tdTomato+ cells in the pancreas. (F) Representative flow cytometry plots of CK19+ cells that co-expressed tdTomato in the pancreas of mice 7 days after injection with tamoxifen. (G) Injection of 12 mg tamoxifen resulted in tdTomato-labeling of 21.8 ± 9.1% of total CK19+ cells. (H) Representative flow cytometry plots of tdTomato + cells that co-expressed CK19 in the pancreas of mice 7 days after injection with tamoxifen. (I) At each tamoxifen dose, ~50% of tdTomato+ cells expressed CK19. (J) Representative image of Mpx1 in tdTomato + cells in acinar tissue. Arrows indicate Mpx1+ tdTomato+ cells. (K,L) 16.8 ± 2.7% of Mpx1+ cells expressed tdTomato, and Mpx1+ cells represented 31.2 ± 21.5% of total tdTomato+ cells by flow cytometry (*n* = 3). (M) Representative image of insulin expression in tdTomato + cells in islet tissue at 7 days after tamoxifen administration. (N, O) 2.2 ± 0.5% of insulin+ cells expressed tdTomato, and insulin+ cells represented 2.0 ± 1.3% of total tdTomato+ cells by flow cytometry (*n* = 5). (P) Representative image of CK19 and insulin associated with tdTomato+ cells. (Q-R) CK19 expression was below the level of detection in tdTomato+ cells with acinar (brackets) and islet localization (arrows). Scale bars = 20μm. Data represent mean ± SD (*P < 0.05).

To explore the direct contribution of CK19+ ductal cells toward beta cell production during islet regeneration following iPan-injection of MSC CdM, we employed the CK19-CreERT Rosa26-mTomato mouse model developed by Means et al^44^ In this temporally controlled lineage tracing model, Cre recombinase was fused to the estrogen receptor and inserted downstream of the CK19 promoter, and Cre was permanently expressed at the plasma membrane in cells expressing CK19 (Figure 1A). In addition, the tdTomato reporter was inserted downstream of a STOP codon flanked by loxP sites at the Rosa26 locus. Upon administration of tamoxifen, Cre recombinase is translocated into the nucleus to cleave loxP sites and excise the STOP codon, resulting in permanent tdTomato expression in CK19+ cells. Tamoxifen was delivered on day −7 and following 1 week, tdT+ and CK19+ cells were visualized in pancreas sections using immunofluorescence microscopy. Labeling efficiency was further quantified at the single cell level by flow cytometry using dissociated pancreas tissue on day 0 (Figure 1B). Notably, administration of tamoxifen always resulted in mosaic labeling of ductal structures (Figure 1C, S1).

To monitor the temporal labeling of CK19+ cells, a dose-response for tamoxifen was first performed (1-12 mg total dose). The optimal delivery of tamoxifen was determined to be 12 mg, administered at 6 mg per day for 2 days, as this dose resulted in the highest detection of total tdTomato+ cells at ~8% of total cells (Figure 1D, E). Out of total CK19+ cells, 21.7 ± 3.2% expressed tdTomato at the 12 mg dose (Figure 1F, G). Furthermore, labeling specificity at 12 mg of tamoxifen, defined as the percentage of tdTomato+ cells co-expressing detectable CK19 was 51.1 ± 14.7%, suggesting other cell types with low CK19 expression were also labeled with tdTomato (Figure 1H, I). Co-staining using an acinar cell antibody (referred to as Mpx1^46^) and an insulin antibody revealed low-level tdTomato labeling in some acinar (Figure 1J) and islet tissue (Figure 1M). Quantification by flow cytometry showed tdTomato co-expression in 16.8 ± 4.7% of Mpx1+ cells (Figure 1K) and Mpx1+ cells represented 31.2 ± 21.5% of total tdTomato+ cells (Figure 1L). In contrast, tdTomato was co-expressed in 2.2 ± 1.1% of insulin+ cells (Figure 1N) and insulin+ cells represented 2.0 ± 1.3% of total tdTomato+ cells (Figure 1O). Importantly, the level of CK19 expression in tdTomato+ cells with acinar and islet localization was below the level of detection by conventional fluorescence microscopy (Figure 1P-R), suggesting the intensity of the tdTomato signal was amplified by the strong Rosa26 promoter in this model.

### CHIR-treated MSC upregulated canonical Wnt signaling

After the generation of Wnt+ CdM and Untreated CdM, the remaining MSC were harvested and analyzed to assess the upregulation of canonical Wnt signaling by assessment of total intracellular beta-catenin by flow cytometry (Supplementary Figure S2A,B) and for nucleus-localized beta-catenin by subcellular fractionation and ELISA (Supplementary Figure S2C). CHIR-treated MSC consistently demonstrated ~1.5-fold increased expression of total intracellular beta-catenin quantified by mean fluorescence intensity (Supplementary Figure S2B) and remarkably, ~10-fold increased nucleus-localized beta-catenin expression (Supplementary Figure S2C), confirming robust activation of canonical Wnt-signaling. Furthermore, CHIR treatment for 24 hours did not alter the MSC phenotype. Greater than 95% of Untreated or CHIR-treated MSC expressed the required stromal markers CD90, CD73, and CD105 and <1% of MSC expressed the hematopoietic markers CD34 or CD45 (Supplementary Figure S2D,E).

### iPan-injection of MSC CdM accelerated islet regeneration in STZ-treated CK19-CreERT Rosa26-mTomato mice using a 21-day protocol

We have previously shown that iPan-injection of Wnt+ MSC CdM on day 10 reduced non-fasted hyperglycemia and increased beta cell mass from days 14 to 42 in non-obese diabetic/severe combined immunodeficiency (NOD/SCID) mice administered 35 mg/kg per day STZ.^27^ In this study, we assessed for the first time whether iPan-injection of CdM could mediate islet regeneration in immune-competent CK19-CreERT Rosa26-mTomato mice. Because the NOD/SCID strain is documented to be sensitive to STZ treatment due to deficiencies in DNA damage response caused by the Prkdc^SCID^ mutation,^48^ we used dose escalation to determine that CK19-CreERT Rosa26-mTomato mice required 50 mg/kg STZ per day (days 0-4) to consistently induce hyperglycemia by day 10 when MSC-CdM was administered (Supplementary Figure S3A,B). Hyperglycemia on day 10 was defined as non-fasted blood glucose levels of 12-25 mmol/L and was achieved in mice receiving 50 mg/kg/day or 60 mg/kg/day STZ (Supplementary Figure S3A,B). Mice administered 40 mg/kg/day STZ did not achieve hyperglycemia over the entire 21-day period. All mice injected with 40, 50, or 60mg/kg/day STZ showed significantly reduced beta cell mass and islet number at day 14 (Supplementary Figure S3C,D). A reduced response to glucose bolus was demonstrated on day 10 (Supplementary Figure S3E,F) and was maintained until day 21 (Supplementary Figure S3G,H) without excessive weight loss (Figure S3I). Thus, 50 mg/kg/day STZ dose was selected for all experiments moving forward.

**Figure 2.**
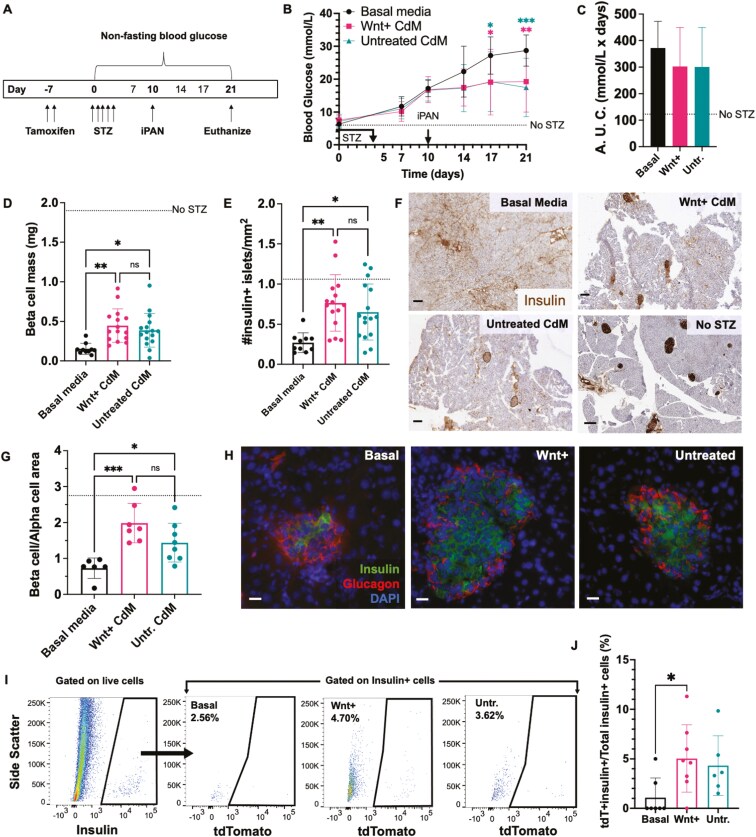
iPan-injection of MSC CdM maintained beta cell mass in CK19-CreERT Rosa26-mTomato mice using a 21-day Protocol. (A) Tamoxifen- (6 mg/day, day −7, −6) and STZ-injected (50 mg/kg/day, days 0-4) CK19-CreERT Rosa26-mTomato female and male mice that were hyperglycemic (> 12 mmol/L) on day 10 were iPan-injected with unconditioned basal media, Wnt+ CdM, or Untreated CdM and non-fasted blood glucose was monitored for 21 days. (B) Blood glucose levels at days 17 and 21 were lower in mice administered Wnt+ CdM (*n* = 15) or Untreated CdM (*n* = 16) compared to mice administered unconditioned basal media (*n* = 12). Dotted line represents glycemia levels in control mice injected with citric acid buffer instead of STZ (no STZ, *n* = 6). (C) Areas under the blood glucose curves were similar between treatment groups. (D,E) Beta cell mass and insulin + islet number/mm^2^ were increased at day 21 in mice administered Wnt+ CdM or Untreated CdM compared to mice administered unconditioned basal media. (F) Representative images of insulin staining in pancreas sections from mice administered unconditioned basal media, Wnt+ CdM, Untreated CdM, or no STZ control. Scale bars = 150 µm. (G) Beta cell/alpha cell area was increased in mice administered Wnt+ CdM or Untreated CdM compared to mice administered unconditioned basal media. (H) Representative images of insulin+ and glucagon+ cells in islets from mice administered Wnt+ CdM, Untreated CdM, or unconditioned basal media. Scale bars = 20 μm. (I) Representative flow cytometry plots of insulin+ cells that co-expressed tdTomato in mice iPan-injected with unconditioned basal media, Wnt+ CdM, or Untreated CdM. (J) At day 21, the frequency of insulin+/tdTomato+ cells was increased in mice that received Wnt+ CdM compared to mice that received unconditioned basal media. Data is shown as mean ± SD (*P < 0.05; *P < 0.01; ***P < 0.001) using matched CdM from a total of *N* = 8 human MSC samples.

Lineage tracing was initiated by administration of 6 mg tamoxifen per day on days −7 and −6 and hyperglycemia was induced by injection of 50 mg/kg/day STZ on days 0-4 in female and male CK19-CreERT Rosa26-mTomato mice. For initial studies, mice randomized to receive iPan-injection with either unconditioned basal media, Wnt+ CdM, or Untreated CdM on day 10, and glycemia was monitored until day 21 (Figure 2A). Mice iPan-injected with either Wnt+ CdM or Untreated CdM showed significantly reduced non-fasted blood glucose levels at days 17 and 21 compared to mice injected with unconditioned basal media (Figure 2B). However, total AUC from days 10 to 21 was not changed between CdM treatment groups versus untreated basal media control (Figure 2C), suggesting longer experiments were required to establish whether functional recovery from hyperglycemia was achieved after CdM-injection compared to unconditioned basal media. Notably, fresh (unfrozen) and matched CdM preparations were used for all 21-day transplantation experiments.

Pancreata harvested on day 21 were sectioned and stained for insulin to determine beta cell mass and islet number. Mice iPan-injected with Wnt+ CdM or Untreated CdM demonstrated increased beta cell mass and insulin+ islet number compared to mice administered unconditioned media (Figure 2D,E). However, mice iPan-injected with Wnt+ CdM (0.45 ± 0.21 mg) or Untreated CdM (0.39 ± 0.21 mg) still demonstrated beta cell mass values that were lower than CAB-injected vehicle control mice (1.9 ± 1.1 mg, Figure S3C, no STZ, dotted line, Figure 2D). Islet number in mice treated with Wnt+ CdM (0.76 ± 0.35 islets/mm^2^) was ~3-fold increased (**P < 0.01) compared to mice administered unconditioned basal media (0.27 ± 0.12 islets/mm^2^) (Figure 2E). Finally, no differences were observed in blood glucose values or beta cell mass when analyzing male or female mice independently in the short-term, 21-day analyses. Representative photomicrographs of pancreas sections from mice iPan-injected with Wnt+ CdM or untreated CdM exhibited more abundant insulin+ islet clusters compared to those treated with basal media (Figure 2F). Collectively, these data suggested that Wnt+ CdM and Untreated CdM delivery resulted in the induction of new beta cell formation and/or protection against continued STZ-induced beta cell deletion after day 10.

Pancreas sections were also immuno-stained for insulin and glucagon to quantify islet beta cell/alpha cell area by immunofluorescent microscopy. Mice iPan-injected with Wnt+ CdM or Untreated CdM exhibited increased beta cell/alpha cell area compared to mice administered unconditioned basal media (Figure 2G). Representative photomicrographs show regenerating islets possessed normal cell distribution /architecture with beta cells situated in the core of the islet and alpha cells at the periphery (Figure 2H).^49^ Notably, significant differences in glucose regulation, beta cell mass, islet number and beta cell/alpha cell area were observed for mice injected with Wnt+ CdM and Untreated CdM compared to Basal media control. Thus, injection of MSC CdM, with or without CHIR-treatment, induced signs of islet regeneration by day 21.

Definitive assessment of the contribution of CK19+ cells to the generation of insulin+ cells after MSC CdM injection requires the detection of insulin-expressing cells at the single cell level. In a separate cohort of mice euthanized at day 21, pancreata (*n* = 6-8 mice/group) were harvested and digested into a single-cell suspension for analyses of insulin and tdTomato co-expression by flow cytometry. iPan-injection injected with unconditioned basal media showed tdTomato expression in only 0.97 ± 0.82% of insulin+ cells indicating conversion of tdTomato+ cells to insulin+ phenotype due to STZ-treatment without MSC CdM supplementation was minimal. In contrast, mice injected with Wnt+ CdM demonstrated > 5-fold increased frequency of insulin+ cells that co-expressed tdTomato (5.04 ± 1.20%, Figure 2I,J), indicating that additional lineage-traced CK19+ cells acquired insulin expression within 11 days of Wnt+ CdM injection. iPan-injection with Untreated CdM similarly demonstrated tdTomato co-expression in 4.32 ± 1.23% of insulin+ cells and reinforced that CK19+ cells contribute to the generation of insulin+ cells after iPan-injection of MSC CdM (Figure 2J). Of note, glucagon and tdTomato co-expression was also analyzed by flow cytometry, the frequency of co-positive events was 50% and unchanged from control samples at day 10 (data not shown).

### iPan-injection of Wnt+ CdM improved glycemic control using a 42-day protocol

**Figure 3.**
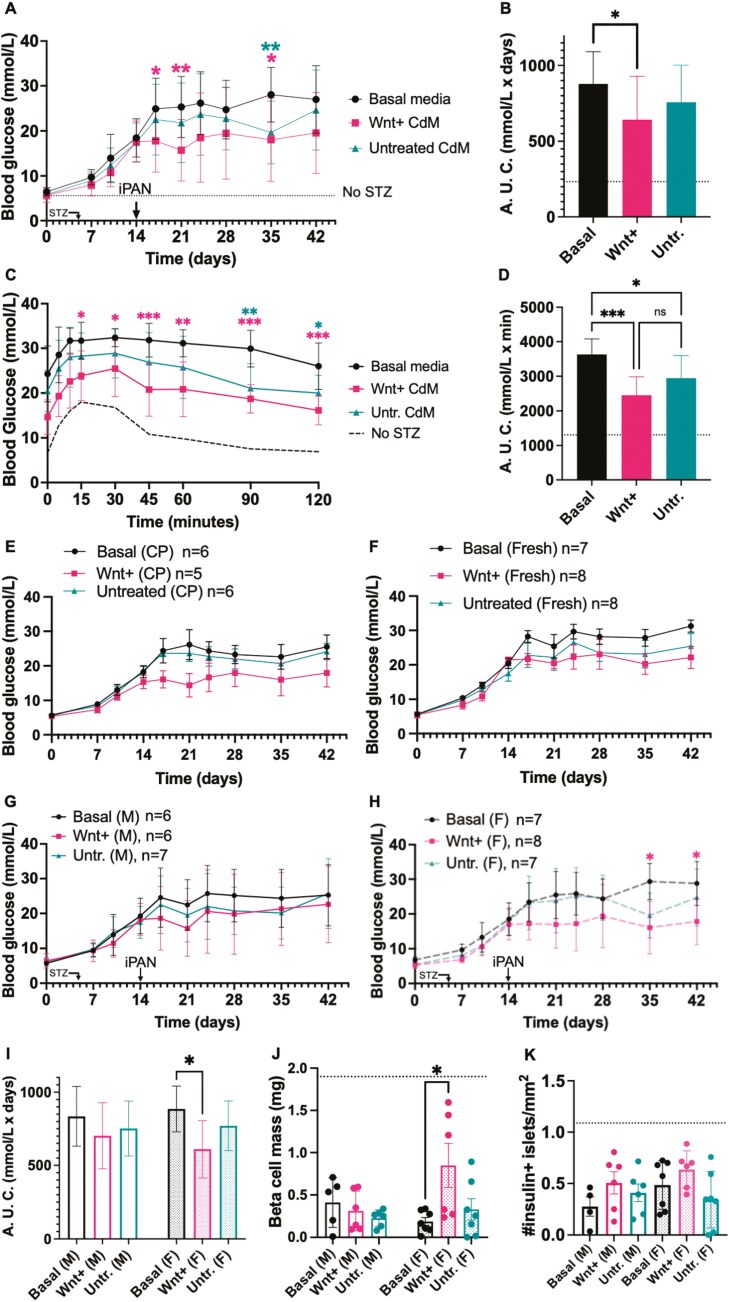
iPan-injection of Wnt+ CdM reduced hyperglycemia and improved glucose tolerance mice using a 42-day protocol. Tamoxifen- (6 mg/day, day −7, −6) and STZ-injected (50 mg/kg/day, days 0-4) female and male CK19-CreERT Rosa26-mTomato mice were iPan-injected with unconditioned basal media (*n* = 16), Wnt+ CdM (*n* = 13), or Untreated CdM (*n* = 13) and non-fasted blood glucose was monitored weekly for 42 days. (A) Blood glucose levels were reduced in mice administered Wnt+ CdM compared to mice injected with unconditioned basal media at days 17, 21, and 35. Dotted lines represent glycemia levels in mice injected with citric acid buffer instead of STZ (no STZ, *n* = 6). (B) Area under the blood glucose curve was lower in mice administered Wnt+ CdM compared to mice administered unconditioned basal media. (C,D) In response to glucose bolus at day 42, mice administered Wnt+ CdM (*n* = 10) and Untreated CdM (*n* = 10) showed improved glucose tolerance compared to mice administered unconditioned basal media (*n* = 10). (E,F) Mice injected with previously cryopreserved CdM showed similar glucose levels compared to mice injected with fresh CdM. (G) Blood glucose levels in male mice were similar between the 3 treatment groups. (H) Blood glucose levels were reduced in female mice administered Wnt+ CdM (*n* = 8) compared to female mice administered unconditioned basal media (*n* = 7). (I) AUC analyses confirmed reduced glucose levels in female mice administered Wnt+ CdM compared to female mice administered unconditioned basal media. (J,K) Beta cell mass was increased in female mice administered Wnt+ CdM compared to female mice administered unconditioned basal media despite no change in total islet number. Data represent mean ± SD (*P < 0.05; **P < 0.01; ***=*P* < 0.001) using matched CdM from a total of *N* = 6 MSC samples.

Because non-fasted blood glucose AUC was equivalent between treatment groups using the 21-day protocol (Figure 2C), and blood glucose levels continued to rise between 10 and 14 days even after MSC-CdM injection in CK19-CreERT Rosa26-mTomato mice (Supplementary Figure S3A), we performed longer-term (42 days) experiments with mice iPan-injected on day 14 rather than day 10. Using this longer protocol, mice injected with Wnt+ CdM demonstrated lower non-fasted glucose levels (at days 17, 21, and 35) compared to mice injected with unconditioned basal media (Supplementary Figure 3A). Importantly, AUC for glucose levels was significantly lowered in mice iPan-injected with Wnt+ CdM compared to basal media injection (Figure 3B).

To assess the ability of mice to respond to elevated glucose levels, glucose tolerance tests were performed at day 42. Mice iPan-injected with Wnt+ CdM or Untreated CdM showed improved ability to respond to a glucose bolus compared to mice iPan-injected with basal media, indicated by lowered AUC for glucose levels (Figure 3C,D). Collectively, enhanced glycemic control was observed in mice iPan-injected with Wnt+ CdM compared to basal media control.

Cryopreserved CdM was used from 2 MSC samples when fresh CdM was not available. A total of 5 mice were injected with cryopreserved Wnt+ CdM and an additional 6 mice with cryopreserved untreated CdM (Supplementary Table S1). Notably, blood glucose throughout the 42-day protocol was statistically equivalent when fresh untreated CdM was compared to cryopreserved untreated CdM, and when fresh Wnt+ CdM was compared to cryopreserved CdM (Figure 3E,F).

To identify sex-specific effects of Wnt+ CdM on glycemic control, a comparison of data from male versus female mice was also conducted. Sub-analyses revealed no discernible difference in non-fasted blood glucose levels between male mice treated with Wnt+ CdM, Untreated CdM, or basal media (Figure 3G). In contrast, female mice that received Wnt+ CdM demonstrated reduced blood glucose levels on days 35 and 42 post-injection (Figure 3H) and area under the curve (Figure 3I) compared to female mice that received basal media, suggesting more pronounced improvement in glycemic control among female mice receiving Wnt+ CdM compared to males. In addition, endpoint analyses of beta cell mass and islet number showed an increased beta cell mass in female mice that received Wnt+ CdM compared to female mice that received basal media (Figure 3J), although no change in islet number was observed (Figure 3K).

### Insulin co-expression in tdTomato+ cells increased following iPan-injection of Wnt+ CdM

**Figure 4.**
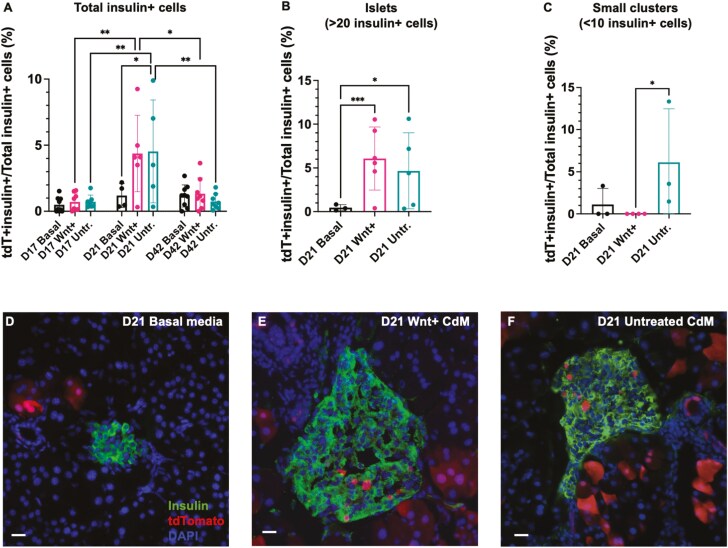
iPan-injection of Wnt+ CdM was associated with increased insulin+/tdTomato+ cells in regenerating islets. Pancreas tissue of female and male CK19-CreERT Rosa26-mTomato mice injected with unconditioned basal media, Wnt+ CdM, or Untreated CdM were analyzed by immunofluorescent microscopy for co-localization of insulin and tdTomato on days 17, 21, and 42. (A) Temporal analyses showed an increase in the frequency of insulin+/tdTomato+ cells occurred between day 17 and 21, and a decrease in the frequency of insulin+/tdTomato+ cells between days 21 and 42. (B) The frequency of insulin+/tdTomato+ cells was increased in larger islets from mice administered Wnt+ CdM or untreated CdM. (C) The frequency of insulin+/tdTomato+ cells was increased in smaller islets from mice administered Untreated CdM compared to mice administered unconditioned basal media. (D-F) Representative images of tdTomato+/insulin+ cells from mice administered basal media, Wnt+ CdM, or Untreated CdM at day 21. Scale bars = 20 μm. Data represent mean ± SD (*=*P* < 0.05, **=*P* < 0.01; ***=*P* < 0.001) using matched CdM from a total of N = 8 MSC samples at day 21 and *N* = 6 MSC samples at day 42.

Time course analyses of pancreas sections detecting insulin+/tdTomato+ lineage-traced cells at days 17, 21, and 42 were performed to provide a temporal and spatial characterization of the contribution of CK19+ lineage-traced cells to islet regeneration. Although no differences were observed between mouse groups injected with Wnt+ CdM or Untreated CdM at day 17 which demonstrated low levels of tdTomato+/insulin+ cells similar to unconditioned basal media-injected control mice, at day 21 the frequency of tdTomato+/insulin+ cells was increased in CdM-injected mice (Figure 4A). However, by day 42 the frequency of tdTomato+/insulin+ cells was diminished in mice injected with Wnt+ CdM or Untreated CdM (Figure 4A), suggesting a contribution of beta cell regeneration from an alternative mechanism may have occurred at later time points. Representative photomicrographs of tdTomato+/insulin+ cells within islets at day 21 are shown in Figure 4D-F.

Previous literature describing islet regeneration often discerns small extra-islet beta cell clusters from larger whole islets as indirect evidence that beta-cell neogenesis had occurred.^50^ Therefore, we segmented our analyses of tdTomato+/insulin+ cells at day 21 based on small islet clusters (< 10 insulin+ cells) versus larger islets (> 20 insulin+ cells). When quantifying tdTomato+/insulin+ cells within larger islets, mice treated with Wnt+ CdM or Untreated CdM demonstrated an increased contribution of tdTomato+ lineage-traced cells compared to mice injected with unconditioned basal media (Figure 4B). In contrast, mice injected with Wnt+ CdM showed a diminished frequency of tdTomato+/insulin+ cells when examining small clusters of < 10 insulin + cells (Figure 4C). Therefore, insulin+/TdTomato+ lineage-traced cells were contained solely in larger, more developed islet structures at 11 days post-injection of Wnt+ CdM.

### Regeneration following iPan-injection of CdM was associated with CK19+ cells within islets

**Figure 5.**
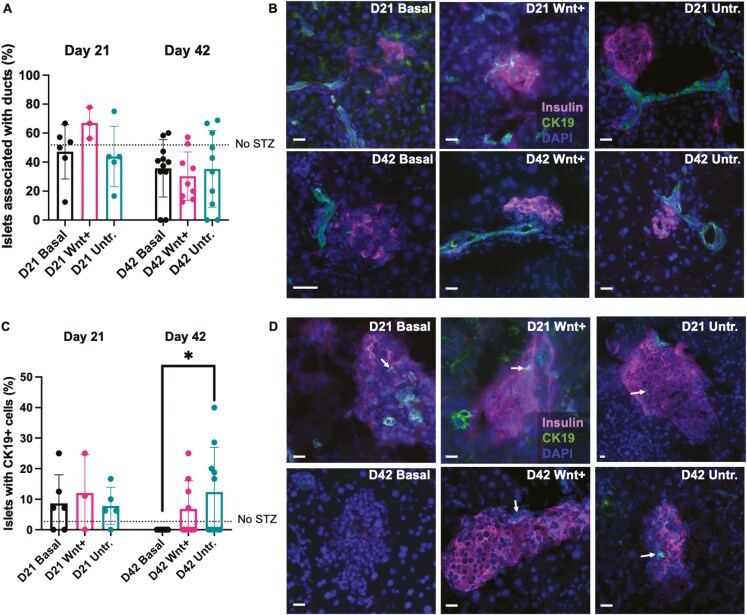
iPan-injection of CdM was associated with CK19+ cells within regenerated islets. Pancreas sections from female and male mice euthanized at day 21 and day 42 were co-stained for insulin and CK19 to determine islet association with ducts and the presence of CK19+ cells within islets. (A) The frequency of islets in direct contact with CK19+ ducts was similar between all groups. Dotted line represents glycemia levels in mice injected with citric acid buffer instead of STZ (no STZ). (B) Representative images of insulin+ islets associated with CK19+ ducts from mice administered unconditioned basal media, Wnt+ CdM, and Untreated CdM at days 21 and 42. (C) At day 42, mice administered Untreated CdM showed a higher frequency of islets containing CK19+ cells compared mice administered unconditioned basal media. (D) Representative images of CK19+ cells within islets (indicated by arrows) from mice administered unconditioned basal media, Wnt+ CdM, and Untreated CdM at day 21 and 42. Scale bars = 20 μm. Data represent mean ± SD (*P < 0.05) using matched CdM from a total of *N* = 8 MSC samples at day 21 and *N* = 6 MSC samples at day 42..

During development in utero, pancreatic acinar and endocrine cells share a common progenitor, Hnf1b+/Nkx6.1+/Sox9+ branching trunk epithelial cells.^50,51^ Our previous studies that performed iPan-injection of Wnt+ CdM in STZ-treated NOD/SCID mice demonstrated that the frequency of regenerating islets in direct contact with CK19+ ductal structures was increased as early as 4 days post-injection (day 14) with Wnt+ CdM.^27^ Studies from other groups have shown the emergence of endocrine cells amongst the ducts following various modalities of pancreatic injury.^52-54^ Therefore, we immuno-stained pancreas sections from both short-term (day 21) and longer-term studies (day 42) for insulin and CK19 to ascertain islet-duct association. Islets were classified as being associated with ducts if an insulin+ islet was in direct contact with a CK19+ duct. Sections from all injected mice demonstrated insulin+ islets in contact with CK19+ ducts, however the frequency of association remained unchanged between CdM and untreated basal media-injected groups (Figure 5A,B). Additionally, Kuljanin *et al*^27^ uniquely observed CK19+ cells within islets after iPan-injection of Wnt+ CdM. Although no differences were seen in the percentage of islets containing CK19+ cells between mice given Wnt+ or Untreated CdM compared to basal media at day 21, by day 42 there was an increase in detectable CK19+ cells within islets in mice administered Untreated CdM compared to unconditioned basal media (Figure 5C,D). Similar to our previous studies in NOD/SCID mice,^27^ an increased frequency of islets containing CK19+ cells was observed in mice that received CdM injection.

### Beta cell proliferation was not increased following iPan-injection of Wnt+ CdM

**Figure 6.**
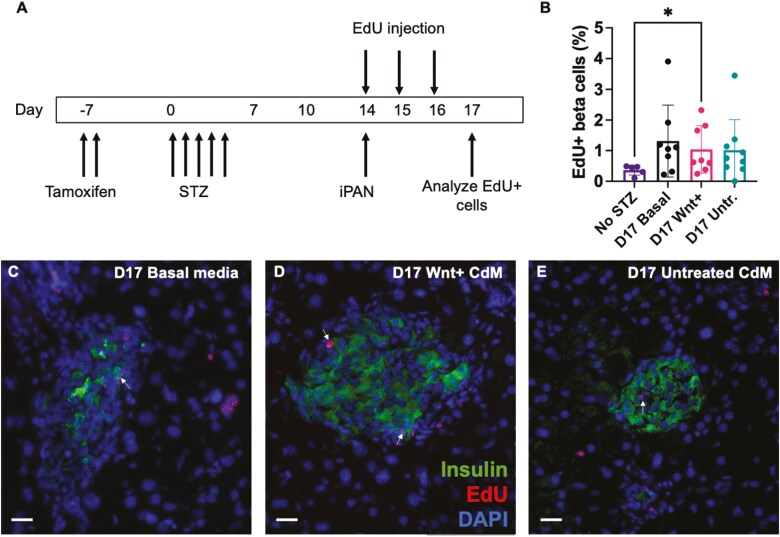
iPan-injection of MSC CdM did not change beta cell proliferation in islets. (A) Tamoxifen- (6mg/day, days −7, −6) and STZ-injected (55 mg/kg/day, days 0-4) female and male CK19-CreERT Rosa26-mTomato mice that were hyperglycemic (> 12 mmol/L) on day 14 were iPan-injected with unconditioned basal media (*n* = 8), Wnt+ CdM (*n* = 8), or Untreated CdM (*n* = 9) and subsequently injected with EdU for 3 consecutive days. (B) There was no difference in the frequency of EdU+/insulin+ cells in mice administered unconditioned basal media, Wnt+ CdM or Untreated CdM. However, compared to citric acid buffer-injected normoglycemic mice (no STZ, *n* = 5), STZ-injected mice administered Wnt+ CdM (*n* = 8) demonstrated an increased frequency of EdU+ beta cells. (C-E) Representative images of EdU+/insulin+ cells (arrows) from mice treated with unconditioned basal media, Wnt+ CdM, or Untreated CdM. Scale bars = 20 μm. Data represent mean ± SD (*P < 0.05) using matched CdM from a total of *N* = 8 MSC samples.

Studies have suggested increased proliferation in surviving beta cell as a predominant mechanism behind increased\beta cell mass following STZ-induced diabetes in rodents.^42,43^ Therefore, we further analyzed pancreas tissue sections for evidence of beta cell proliferation. In Kuljanin et al, we previously used EdU incorporation to observe an increase in proliferating insulin+ cells as soon as 1 day following iPan-injection of Wnt+ CdM in STZ-treated NOD/SCID mice, however, beta cell proliferation returned to baseline levels by 4 days post-injection.^27^ Following the iPan-injection of mice, we injected with EdU once daily for 3 days and analyzed regenerating islets for proliferating beta cells via insulin and EdU staining (Figure 6A). The frequency of EdU+/insulin+ cells within islets without STZ treatment was typically < 0.5%. Mice treated with STZ and subsequently iPan-injected with unconditioned basal media, Wnt+ CdM, and untreated CdM demonstrated equal levels of EdU+/insulin+ cells (~1%) and suggested that a small (2-fold) increase of beta-cell proliferation was mediated primarily by STZ-injection rather than iPan-injection of MSC CdM (Figure 6B). Nonetheless, treatment with STZ followed by Wnt+ CdM resulted in an increase in dividing beta cells compared to control mice not given STZ (Figure 6B). Representative photomicrographs of EdU+ cells within islets are shown in Figure 6C-E. Finally, EdU incorporation into non-islet cells with acinar or ductal morphology was rarely observed at 3 days post iPan-injection.^27^

## Discussion

In this study iPan-injection of Wnt+ CdM into multiple low-dose STZ-treated CK19-CreERT Rosa26-mTomato lowered hyperglycemia, augmented glucose tolerance, increased the number of islets per mm^2^ and improved beta cell mass compared to Basal media-injected control mice. We also demonstrated, for the first time, that some insulin+ cells originated from a CK19+ cell during regeneration stimulated by Wnt+ MSC CdM. However, only 5% of insulin+ cells were co-labelled with tdTomato, indicating that other mechanisms originating from an alternate and undetermined cell type also occurred.

These studies, for the first time, employed iPan-injection of Wnt+ CdM into fully immune-competent mice and was sufficient to lower non-fasted blood glucose levels and increase the ability to regulate a glucose bolus without delivery of the source MSC. Qualitatively, islet regeneration was similar to our previously reported studies injecting MSC CdM into immunodeficient NOD/SCID mice using multiple low-dose STZ treatment, a model originally designed for the transfer of islet regenerative human cell populations.^19^ However, the employed CK19 mouse is fully immune-competent, and therefore infiltration of immune cells was expected after STZ treatment.^55^ Because MSCs are recognized to have immune-modulatory properties, the attenuation of hyperglycemia or the slowing of diabetes progression may include immune inhibition effects of MSC CdM on infiltrating T-cell function.^17^ It cannot be determined from this study whether higher beta cell mass and islet number after treatment with MSC CdM were due to the suppression of continued damage mediated by infiltrating immune cells after STZ treatment. Experiments utilizing MSC-CdM injection to address potential immunomodulatory effects by the MSC-CdM in models of autoimmune diabetes (NOD mouse) are currently underway.

With a focus on the day 42 experiments, reduced non-fasted blood glucose levels by AUC and enhanced glucose regulation evaluated by glucose tolerance tests were only observed in mice given Wnt+ CdM compared to Basal media-injected controls. Thus, activation of the Wnt-pathway, using the GSK inhibitor, CHIR99021 was required to observe consistent improvements in glucose control using this lineage tracing model. In human MSC, activation of the Wnt-pathway is primarily involved in cell growth and fate decisions. We have previously analyzed the secretome of Untreated MSC CdM and found that highly regenerative BM-MSC lines had upregulated protein secretion of known targets of canonical Wnt/beta-catenin signaling.^24,25^ Stimulation of the canonical Wnt-signaling pathway has been shown to enhance the release of islet regenerative proteins in non-regenerative BM-MSC lines, defined by their ability to reduce glucose levels in STZ-treated mice.^24-27^ However, mice administered Wnt+ CdM in this study did not consistently show significantly reduced glucose levels, improved glucose tolerance, or increased beta cell mass compared to control mice than mice given Untreated CdM. Although Wnt+ CdM may provide extra regenerative signals downstream of Wnt-pathway transcriptional regulation to enhance beta cell regeneration, thorough proteomic and gene expression comparison of Wnt+ CdM and Untreated CdM is necessary and warranted for potential biotherapeutic drug discovery initiatives.

The CK19-CreERT lineage tracing mouse developed by Means et al in 2008 was crossed with a Rosa26-mTomato reporter mouse to provide lineage tracing of CK19 cells in this study.^44^ At 11 days following iPan-injection of Wnt+ CdM (day 21), tdTomato+/insulin+ co-expressing cells were increased to 5-fold higher compared to controls given unconditioned basal media. However, tamoxifen treatment (12 mg total) in CK19-CreERT Rosa26-mTomato mice resulted in the mosaic labeling of approximately half of the pancreatic ductal cells, and significant TdTomato-labelling was also observed in 17% of acinar cells. Therefore, we cannot rule out the possibility that regenerated insulin + was not derived from the acinar cell lineage. CK19-CreERT Rosa26-mTomato mice given corn oil only (no tamoxifen) did not demonstrate any tdTomato expression. Labeling of cells outside the CK19 lineage may be due to Rosa26-mTomato mice having increased sensitivity to Cre compared to the Rosa26-YFP mice previously used in similar studies.^44,45^ Furthermore, antibody staining showed expression of CK19 was restricted to the ductal epithelium, though it is possible that some acinar cells endogenous low expression of CK19 resulting in Cre-mediated expression of tdTomato. In 2019, Kanayama et al, characterized a CK19-iCre lineage tracing mouse crossed to the same Rosa26-mTomato reporter mouse and also found iCre mRNA and tdTomato expression in both ductal and acinar cells. However, the percentage of labeled acinar and ductal cells were not quantified.^56^ Given the low percentage of islets in contact with CK19+ ducts and the relatively low frequency of tdTomato+/insulin+ cells observed in this study, CK19+ cells were not the only cell type that contributed to beta cell regeneration in this model. The search for alternate sources of beta cells generated after Wnt+ CdM injection includes the tracing of alpha cells in Glucagon-CreERT Rosa26-mTomato mice.

## Conclusions

Stimulating endogenous regeneration using protein effectors derived from BM-MSC grown under Wnt pathway stimulatory conditions has proven to be a promising approach to replenish beta cells depleted in STZ-treated mice. Given the controversy surrounding the occurrence of beta cell neogenesis from precursor cells in the adult pancreas (ductal, acinar, alpha cells, delta cells), the investigation into other cellular mechanisms by which Wnt+ CdM stimulated beta cell regeneration and identification of the cell source of regenerating beta cells is warranted. This study identified CK19+ to insulin+ cell conversion as a potential mechanism behind BM-MSC CdM-induced islet regeneration. We showed that iPan injection of Wnt+ CdM consistently stimulated beta cell regeneration in the presence of an active immune system with aggressive STZ-mediated beta cell ablation (50 mg/kg/day). Lineage tracing of CK19+ cells revealed ductal and/or acinar cells provided a minor contribution to beta cell regeneration induced by Wnt+ CdM, leaving the possibility of other regenerative mechanisms, such as the induction of alpha-to-beta cell transition, to be explored in future studies.

## Supplementary Material

sxaf036_suppl_Supplementary_Figures_S2-S3_Table_S1

## Data Availability

The data that support the findings of this study are available from the corresponding author upon reasonable request.
