# Supplementary material for: Contribution of cytokeratin 19-expressing cells towards islet regeneration induced by multipotent stromal cell secreted proteins
Source: Stem Cells. 2025 Jun 10;43(9):sxaf036. doi: 10.1093/stmcls/sxaf036 (PMC12371234; doi:10.1093/stmcls/sxaf036)
Supplement: sxaf036_suppl_Supplementary_Figures_S2-S3_Table_S1 [file sxaf036_suppl_supplementary_figures_s2-s3_table_s1.pptx]

## Slide 1
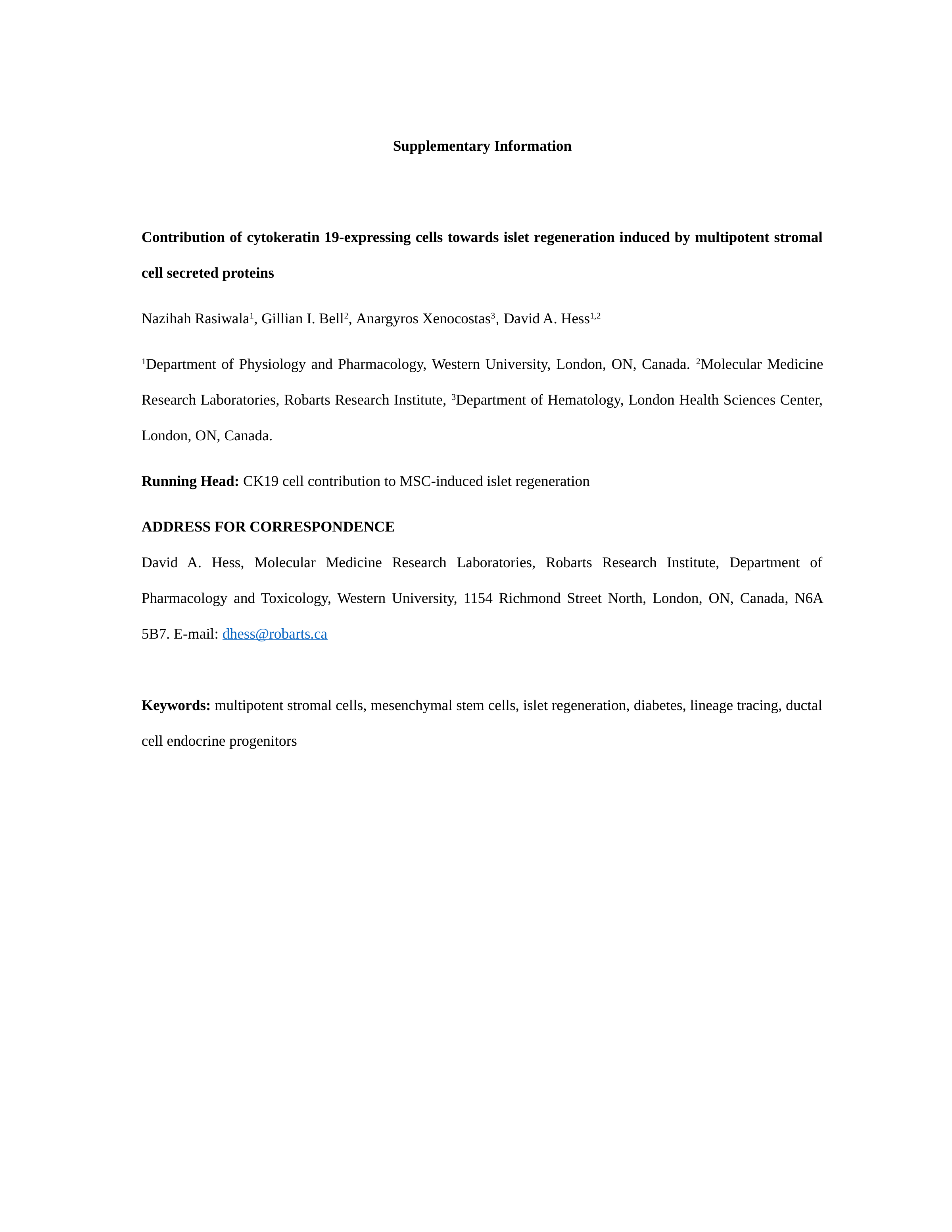

Supplementary Information
Contribution of cytokeratin 19-expressing cells towards islet regeneration induced by multipotent stromal cell secreted proteins
Nazihah Rasiwala1, Gillian I. Bell2, Anargyros Xenocostas3, David A. Hess1,2
1Department of Physiology and Pharmacology, Western University, London, ON, Canada. 2Molecular Medicine Research Laboratories, Robarts Research Institute, 3Department of Hematology, London Health Sciences Center, London, ON, Canada.
Running Head: CK19 cell contribution to MSC-induced islet regeneration
Address for Correspondence
David A. Hess, Molecular Medicine Research Laboratories, Robarts Research Institute, Department of Pharmacology and Toxicology, Western University, 1154 Richmond Street North, London, ON, Canada, N6A 5B7. E-mail: dhess@robarts.ca
Keywords: multipotent stromal cells, mesenchymal stem cells, islet regeneration, diabetes, lineage tracing, ductal cell endocrine progenitors

## Slide 2
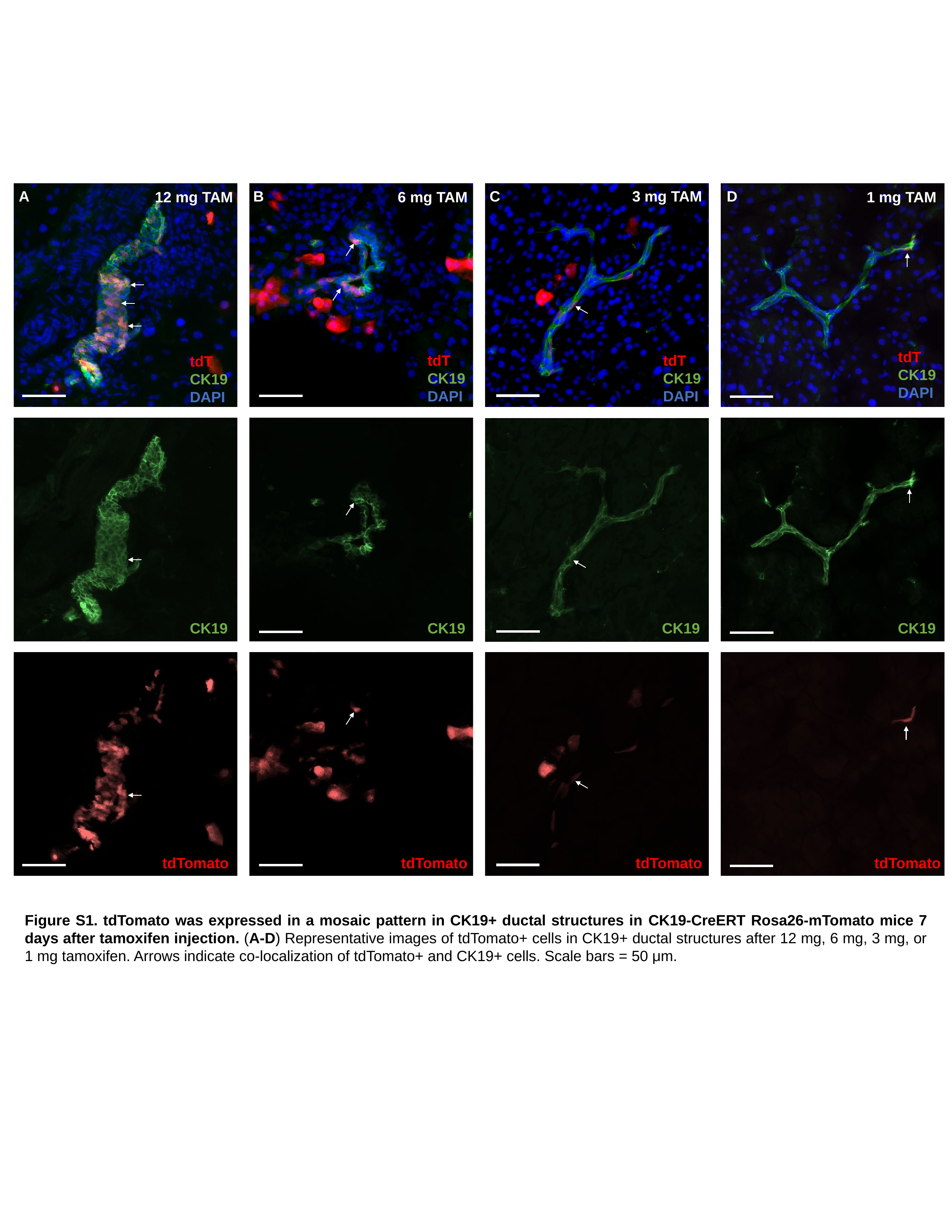

A
C
B
D
3 mg TAM
12 mg TAM
6 mg TAM
1 mg TAM
tdT
CK19
DAPI
tdT
CK19
DAPI
tdT
CK19
DAPI
tdT
CK19
DAPI
CK19
CK19
CK19
CK19
tdTomato
tdTomato
tdTomato
tdTomato
Figure S1. tdTomato was expressed in a mosaic pattern in CK19+ ductal structures in CK19-CreERT Rosa26-mTomato mice 7 days after tamoxifen injection. (A-D) Representative images of tdTomato+ cells in CK19+ ductal structures after 12 mg, 6 mg, 3 mg, or 1 mg tamoxifen. Arrows indicate co-localization of tdTomato+ and CK19+ cells. Scale bars = 50 μm.

## Slide 3
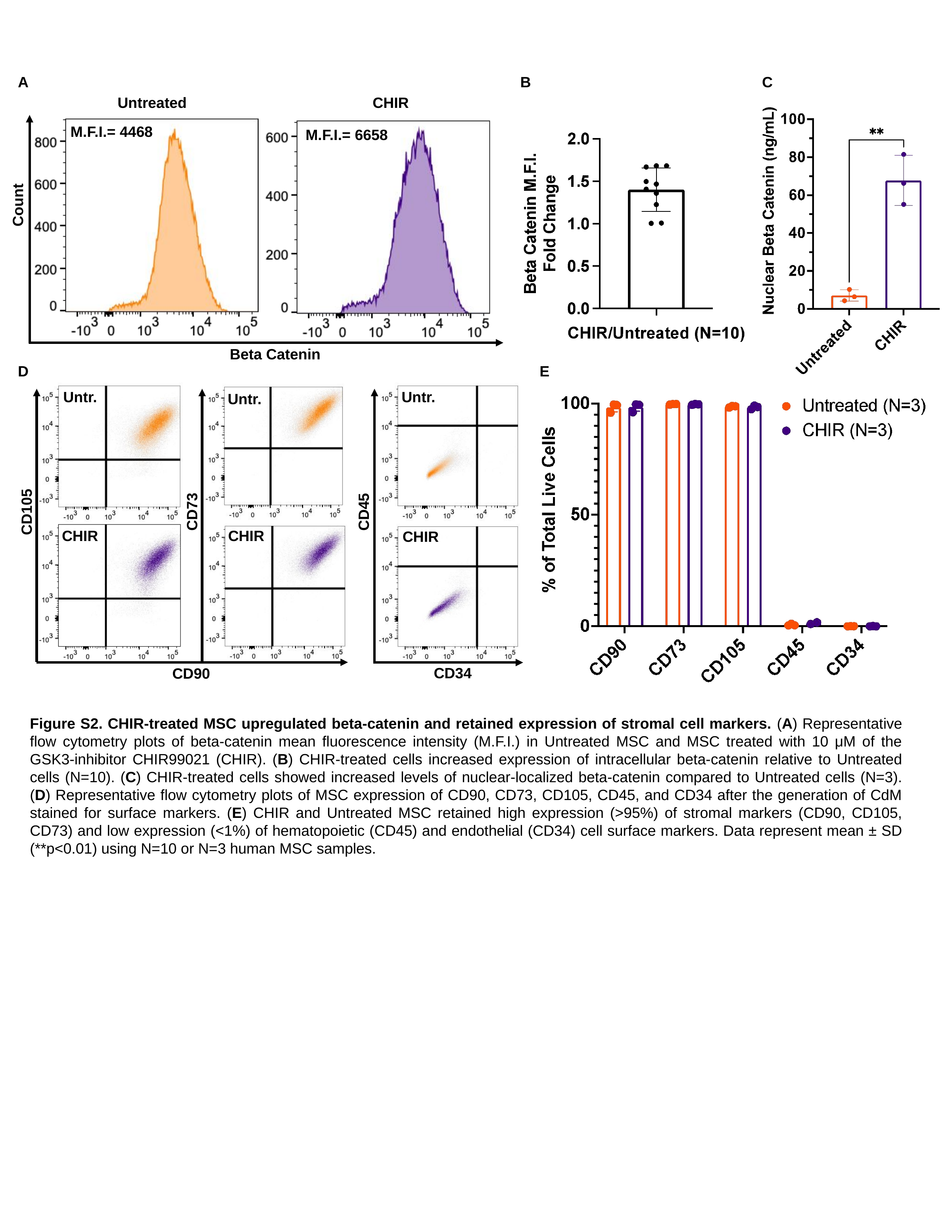

A
B
C
Untreated
CHIR
M.F.I.= 4468
M.F.I.= 6658
Count
Beta Catenin
D
E
Untr.
Untr.
Untr.
CD105
CD73
CD45
CHIR
CHIR
CHIR
CD34
CD90
Figure S2. CHIR-treated MSC upregulated beta-catenin and retained expression of stromal cell markers. (A) Representative flow cytometry plots of beta-catenin mean fluorescence intensity (M.F.I.) in Untreated MSC and MSC treated with 10 μM of the GSK3-inhibitor CHIR99021 (CHIR). (B) CHIR-treated cells increased expression of intracellular beta-catenin relative to Untreated cells (N=10). (C) CHIR-treated cells showed increased levels of nuclear-localized beta-catenin compared to Untreated cells (N=3). (D) Representative flow cytometry plots of MSC expression of CD90, CD73, CD105, CD45, and CD34 after the generation of CdM stained for surface markers. (E) CHIR and Untreated MSC retained high expression (>95%) of stromal markers (CD90, CD105, CD73) and low expression (<1%) of hematopoietic (CD45) and endothelial (CD34) cell surface markers. Data represent mean ± SD (**p<0.01) using N=10 or N=3 human MSC samples.

## Slide 4
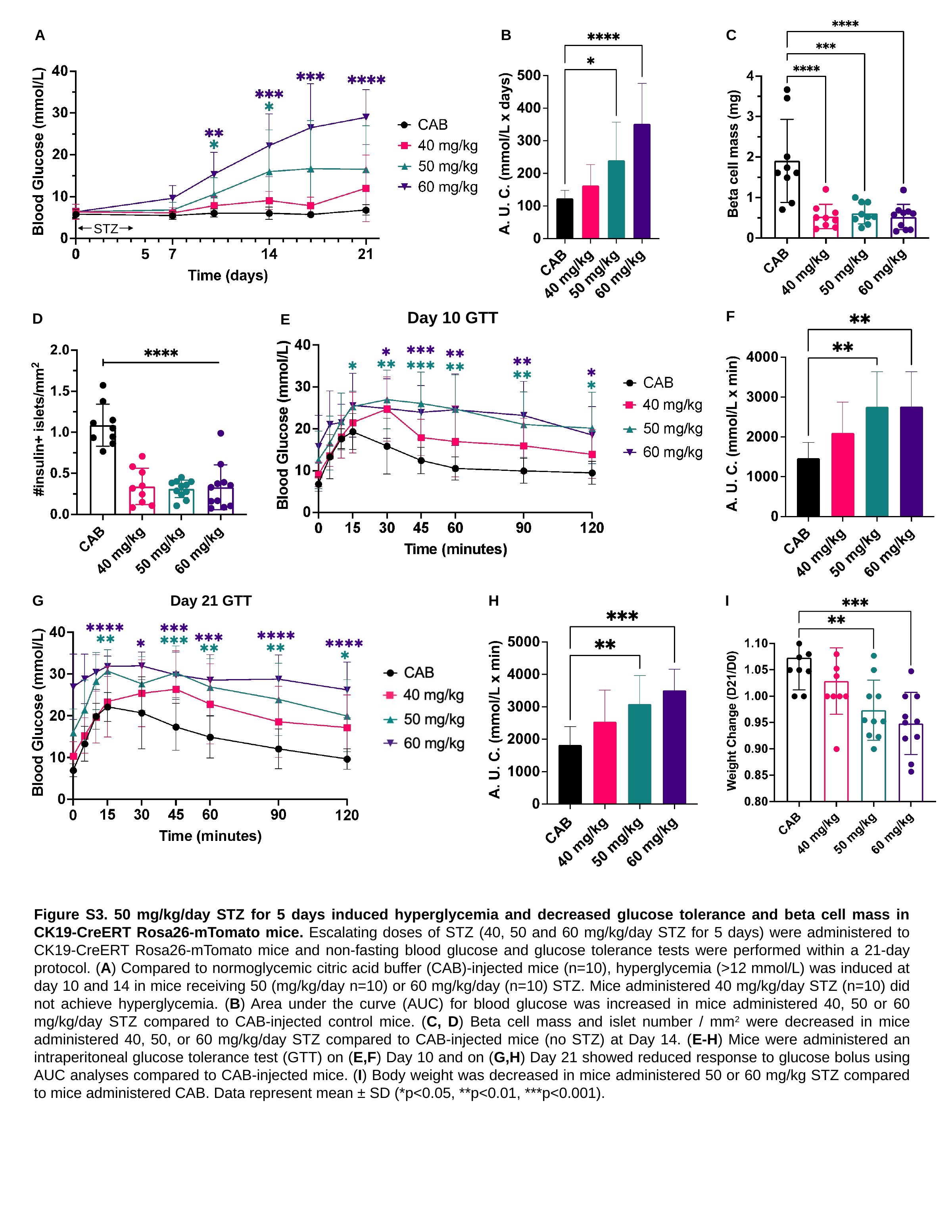

A
B
C
STZ
5
F
Day 10 GTT
D
E
✱✱✱
✱
✱✱
✱✱
✱✱
✱✱✱
✱
✱✱
✱
✱✱
✱
G
Day 21 GTT
H
I
✱✱✱✱
✱✱✱
✱✱✱✱
✱✱✱
✱✱
✱✱✱
✱
✱✱✱✱
✱✱
✱✱
✱
Figure S3. 50 mg/kg/day STZ for 5 days induced hyperglycemia and decreased glucose tolerance and beta cell mass in CK19-CreERT Rosa26-mTomato mice. Escalating doses of STZ (40, 50 and 60 mg/kg/day STZ for 5 days) were administered to CK19-CreERT Rosa26-mTomato mice and non-fasting blood glucose and glucose tolerance tests were performed within a 21-day protocol. (A) Compared to normoglycemic citric acid buffer (CAB)-injected mice (n=10), hyperglycemia (>12 mmol/L) was induced at day 10 and 14 in mice receiving 50 (mg/kg/day n=10) or 60 mg/kg/day (n=10) STZ. Mice administered 40 mg/kg/day STZ (n=10) did not achieve hyperglycemia. (B) Area under the curve (AUC) for blood glucose was increased in mice administered 40, 50 or 60 mg/kg/day STZ compared to CAB-injected control mice. (C, D) Beta cell mass and islet number / mm2 were decreased in mice administered 40, 50, or 60 mg/kg/day STZ compared to CAB-injected mice (no STZ) at Day 14. (E-H) Mice were administered an intraperitoneal glucose tolerance test (GTT) on (E,F) Day 10 and on (G,H) Day 21 showed reduced response to glucose bolus using AUC analyses compared to CAB-injected mice. (I) Body weight was decreased in mice administered 50 or 60 mg/kg STZ compared to mice administered CAB. Data represent mean ± SD (*p<0.05, **p<0.01, ***p<0.001).

## Slide 5
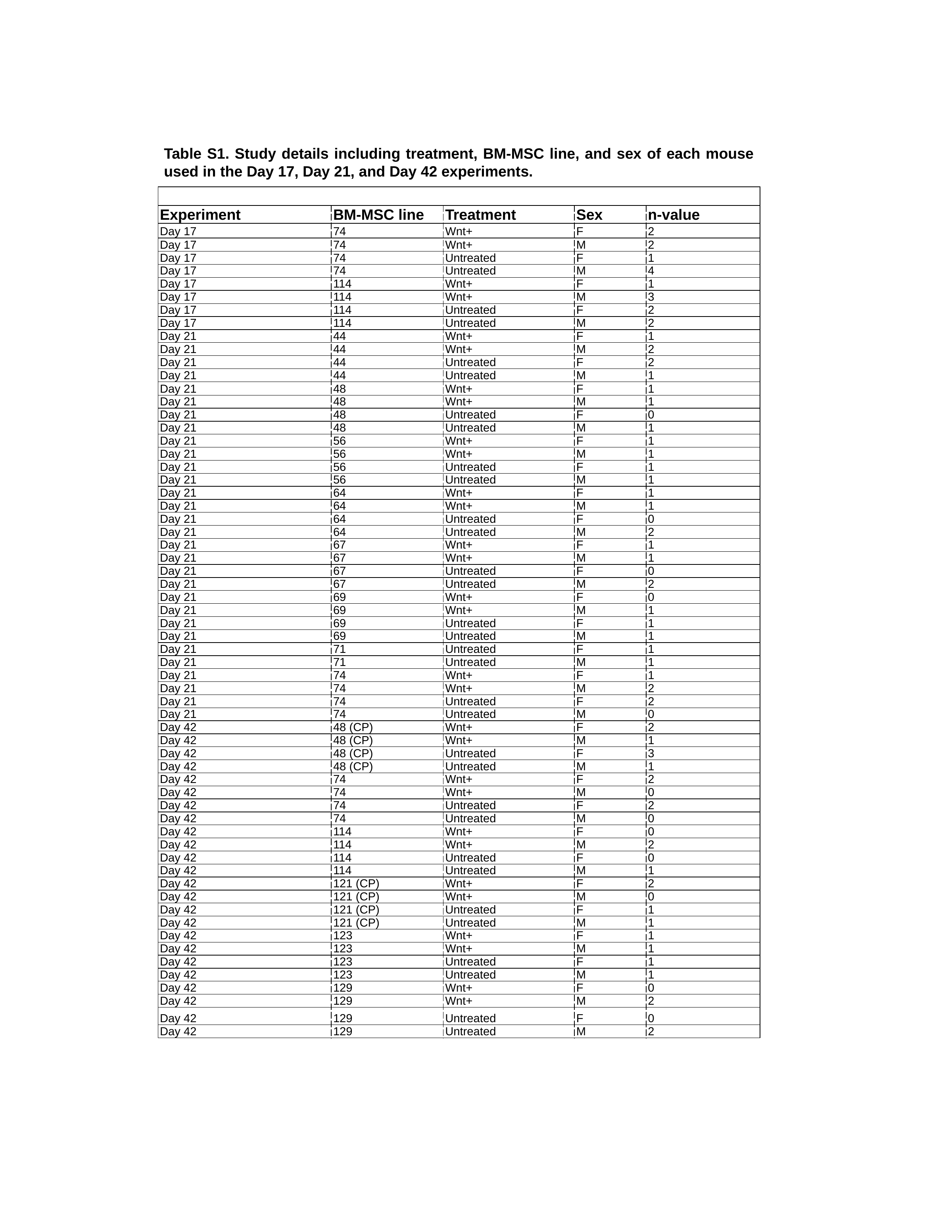

Table S1. Study details including treatment, BM-MSC line, and sex of each mouse used in the Day 17, Day 21, and Day 42 experiments.
| | | | | |
| --- | --- | --- | --- | --- |
| Experiment | BM-MSC line | Treatment | Sex | n-value |
| Day 17 | 74 | Wnt+ | F | 2 |
| Day 17 | 74 | Wnt+ | M | 2 |
| Day 17 | 74 | Untreated | F | 1 |
| Day 17 | 74 | Untreated | M | 4 |
| Day 17 | 114 | Wnt+ | F | 1 |
| Day 17 | 114 | Wnt+ | M | 3 |
| Day 17 | 114 | Untreated | F | 2 |
| Day 17 | 114 | Untreated | M | 2 |
| Day 21 | 44 | Wnt+ | F | 1 |
| Day 21 | 44 | Wnt+ | M | 2 |
| Day 21 | 44 | Untreated | F | 2 |
| Day 21 | 44 | Untreated | M | 1 |
| Day 21 | 48 | Wnt+ | F | 1 |
| Day 21 | 48 | Wnt+ | M | 1 |
| Day 21 | 48 | Untreated | F | 0 |
| Day 21 | 48 | Untreated | M | 1 |
| Day 21 | 56 | Wnt+ | F | 1 |
| Day 21 | 56 | Wnt+ | M | 1 |
| Day 21 | 56 | Untreated | F | 1 |
| Day 21 | 56 | Untreated | M | 1 |
| Day 21 | 64 | Wnt+ | F | 1 |
| Day 21 | 64 | Wnt+ | M | 1 |
| Day 21 | 64 | Untreated | F | 0 |
| Day 21 | 64 | Untreated | M | 2 |
| Day 21 | 67 | Wnt+ | F | 1 |
| Day 21 | 67 | Wnt+ | M | 1 |
| Day 21 | 67 | Untreated | F | 0 |
| Day 21 | 67 | Untreated | M | 2 |
| Day 21 | 69 | Wnt+ | F | 0 |
| Day 21 | 69 | Wnt+ | M | 1 |
| Day 21 | 69 | Untreated | F | 1 |
| Day 21 | 69 | Untreated | M | 1 |
| Day 21 | 71 | Untreated | F | 1 |
| Day 21 | 71 | Untreated | M | 1 |
| Day 21 | 74 | Wnt+ | F | 1 |
| Day 21 | 74 | Wnt+ | M | 2 |
| Day 21 | 74 | Untreated | F | 2 |
| Day 21 | 74 | Untreated | M | 0 |
| Day 42 | 48 (CP) | Wnt+ | F | 2 |
| Day 42 | 48 (CP) | Wnt+ | M | 1 |
| Day 42 | 48 (CP) | Untreated | F | 3 |
| Day 42 | 48 (CP) | Untreated | M | 1 |
| Day 42 | 74 | Wnt+ | F | 2 |
| Day 42 | 74 | Wnt+ | M | 0 |
| Day 42 | 74 | Untreated | F | 2 |
| Day 42 | 74 | Untreated | M | 0 |
| Day 42 | 114 | Wnt+ | F | 0 |
| Day 42 | 114 | Wnt+ | M | 2 |
| Day 42 | 114 | Untreated | F | 0 |
| Day 42 | 114 | Untreated | M | 1 |
| Day 42 | 121 (CP) | Wnt+ | F | 2 |
| Day 42 | 121 (CP) | Wnt+ | M | 0 |
| Day 42 | 121 (CP) | Untreated | F | 1 |
| Day 42 | 121 (CP) | Untreated | M | 1 |
| Day 42 | 123 | Wnt+ | F | 1 |
| Day 42 | 123 | Wnt+ | M | 1 |
| Day 42 | 123 | Untreated | F | 1 |
| Day 42 | 123 | Untreated | M | 1 |
| Day 42 | 129 | Wnt+ | F | 0 |
| Day 42 | 129 | Wnt+ | M | 2 |
| Day 42 | 129 | Untreated | F | 0 |
| Day 42 | 129 | Untreated | M | 2 |
